# Nodular fasciitis of the breast: the report of three cases

**DOI:** 10.1186/s12905-022-01631-2

**Published:** 2022-03-03

**Authors:** Wanling Lin, Lingyun Bao

**Affiliations:** Department of Ultrasound, Affiliated Hangzhou First People’s Hospital, Zhejiang University School of Medicine, 261 Huansha Rd, Shangcheng District, Hangzhou, 310006 Zhejiang China

**Keywords:** Nodular fasciitis, Breast, Ultrasonography

## Abstract

**Background:**

Nodular Fasciitis is a benign fibroblastic proliferation in soft tissues, which mostly occurs in the upper extremities, trunk, head and neck region. It is rarely reported to occur in the breast.

**Case presentation:**

Herein, we present sonograms of nodular fasciitis in the breast at different durations in three cases. In Case 1, we provided the longest follow-up time in all literatures. In Case 2 and Case 3, we provided the automated breast ultrasound finding of breast nodular fasciitis for the first time.

**Conclusion:**

Nodular Fasciitis shows clinical features and ultrasonography findings are similar to those of breast cancer. For superficially located breast lesions with a single and rapid growth, nodular fasciitis may be considered in the differential diagnosis of benign entities resembling malignant tumors on breast imaging.

## Background

Nodular fasciitis (NF) is a benign reactive proliferative lesion of fibroblasts that can occur throughout the body [1]. The most common site of NF is the upper limbs, followed by the head, neck and trunk [2]. The most typical features are the sudden appearance and rapid growth of palpable lesions [3]. However, it has rarely been reported to occur in the breast. NF can mimic breast cancer clinically, radiologically, and histopathologically. We present three cases of NF in the breast.

## Case presentation

### Case no. 1

**Fig. 1:**
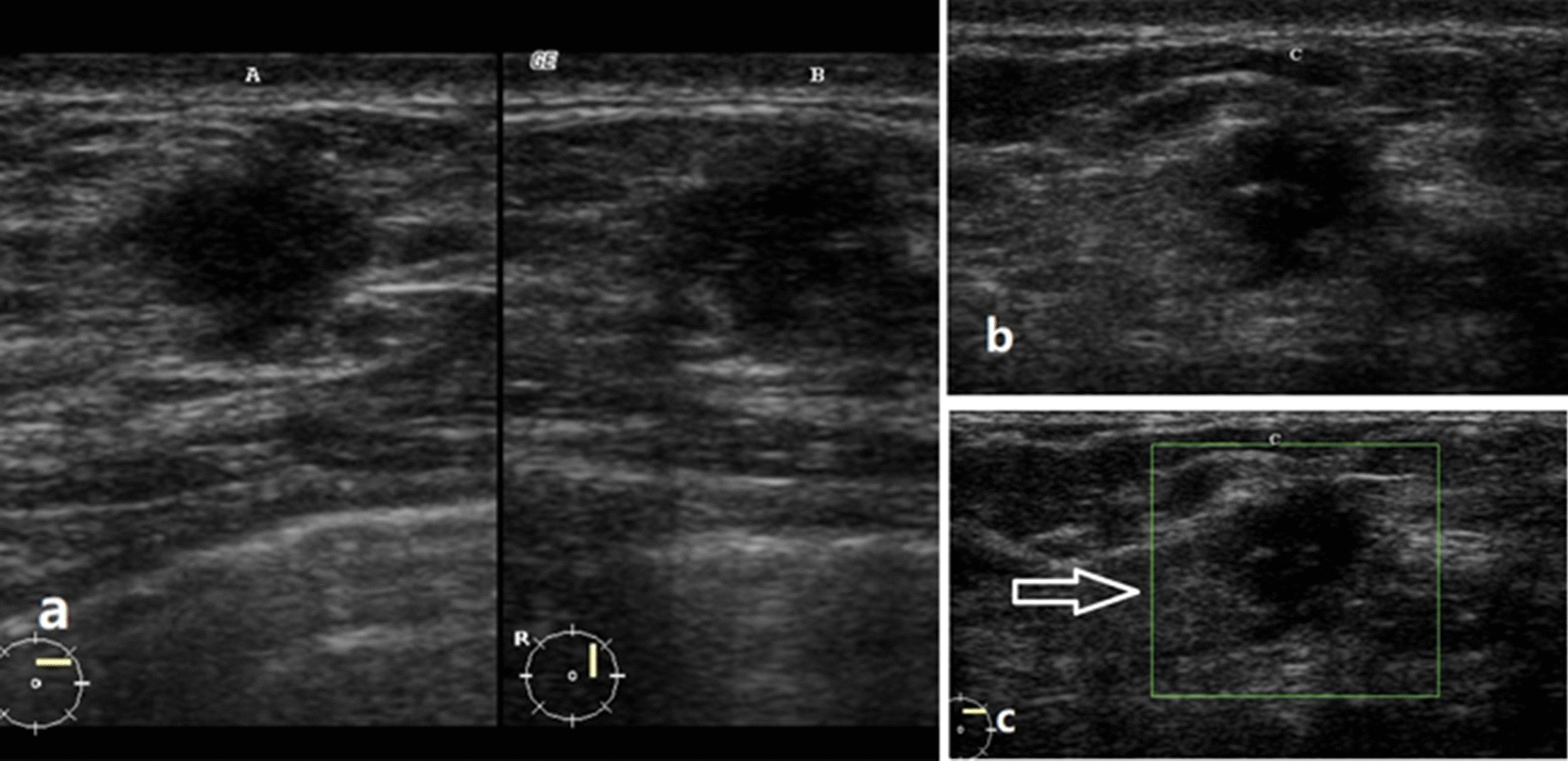
**a**, **b** transverse, sagittal and coronal US showed the lesion at 1 o 'clock in the right breast. It was a 10 × 10 × 8 mm irregular, indistinct and heterogeneous hypoechoic mass, which had no posterior shadowing. **c** Color Doppler shows no vascularity in the mass (white arrows)

A 43-year-old woman presented to the hospital because of a swelling mass in her right breast of 8 months’ duration. Through physical examination, a firm mobile 2 × 3 cm mass was detected at the 1-o’clock position of the right breast. There was no tenderness on palpation, and the patient showed no covering skin changes or nipple discharge. Axillary and supraclavicular lymph nodes were not touched. The patient denied any recent trauma and had no family history of breast cancer. The handheld breast ultrasonography (US) examination (Fig. 1) revealed that a lesion was located at the 1 o’clock position of the right breast. It was a 10 × 10 × 8 mm, irregular, indistinct and heterogeneous hypoechoic mass, which had no posterior shadowing. The lesion showed no vascularity on Dopple US examination. Subsequently,an excisional biopsy was performed. Histopathologic features were diagnosed as NF. At the most recent follow-up, twelve years postoperatively, no recurrence was observed in the case.

### Case no. 2

**Fig. 2:**
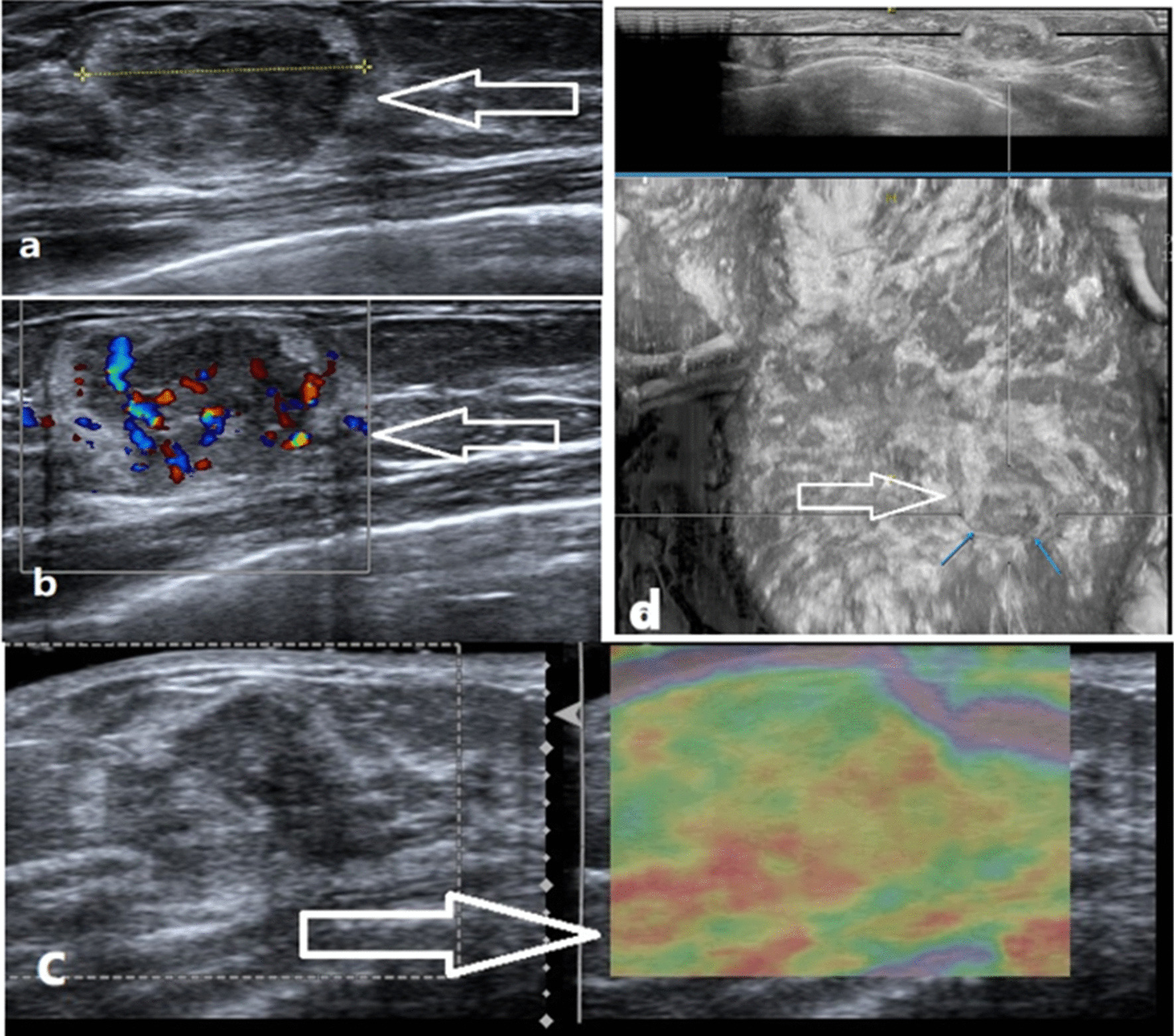
**a** The hand-held ultrasound revealed an irregular and uneven 22 × 13 × 21 mm hypoechoic mass at 3 o 'clock in the right breast with an echo halo extending from the anterior edge of the breast area to the peribreast adipose layer (white arrow). **b** Color Doppler imaging showed some vascular distribution in the mass(white arrows). **c** Ultrasound elastography showed a Tsukuba stiffness score of 3 of the lesion (white arrows). **d** By ABUS, the lesion showed an irregular shape, an indistinct margin, heterogeneous hypoechoic with an echogenic halo (white arrows)

A 37-year-old Asian woman presented with a palpable, painless mass in the right lower inner breast for 15 days. There was no history of trauma. She had no family history of breast cancer. The handheld and automated breast ultrasound (ABUS) examination (Fig. 2) revealed that a lesion was located 30 mm from the nipple at the 3 o’clock position in the right breast and was surrounded by subcutaneous adipose tissue, with attachments to the anterior mammary fascia. It was a 22 × 13 × 21-mm, irregular, indistinct, without associated calcification, heterogeneous hypoechoic mass which was surrounded by a faint halo of high echo and posterior enhancement. The lesion and the surroundings showed some vascular distribution on Dopple US examination (Fig. 2). The elastography revealed a Tsukuba stiffness score of 3 of the lesion (Fig. 2). The core biopsy of the lesion was then performed with a 16 gauge needle. Although tumor spindle cell proliferation was suspected, no definite diagnosis was made. Finally, the percutaneous removal of the mass was performed. The pathologic findings led to a diagnosis of NF.

### Case no. 3

**Fig. 3:**
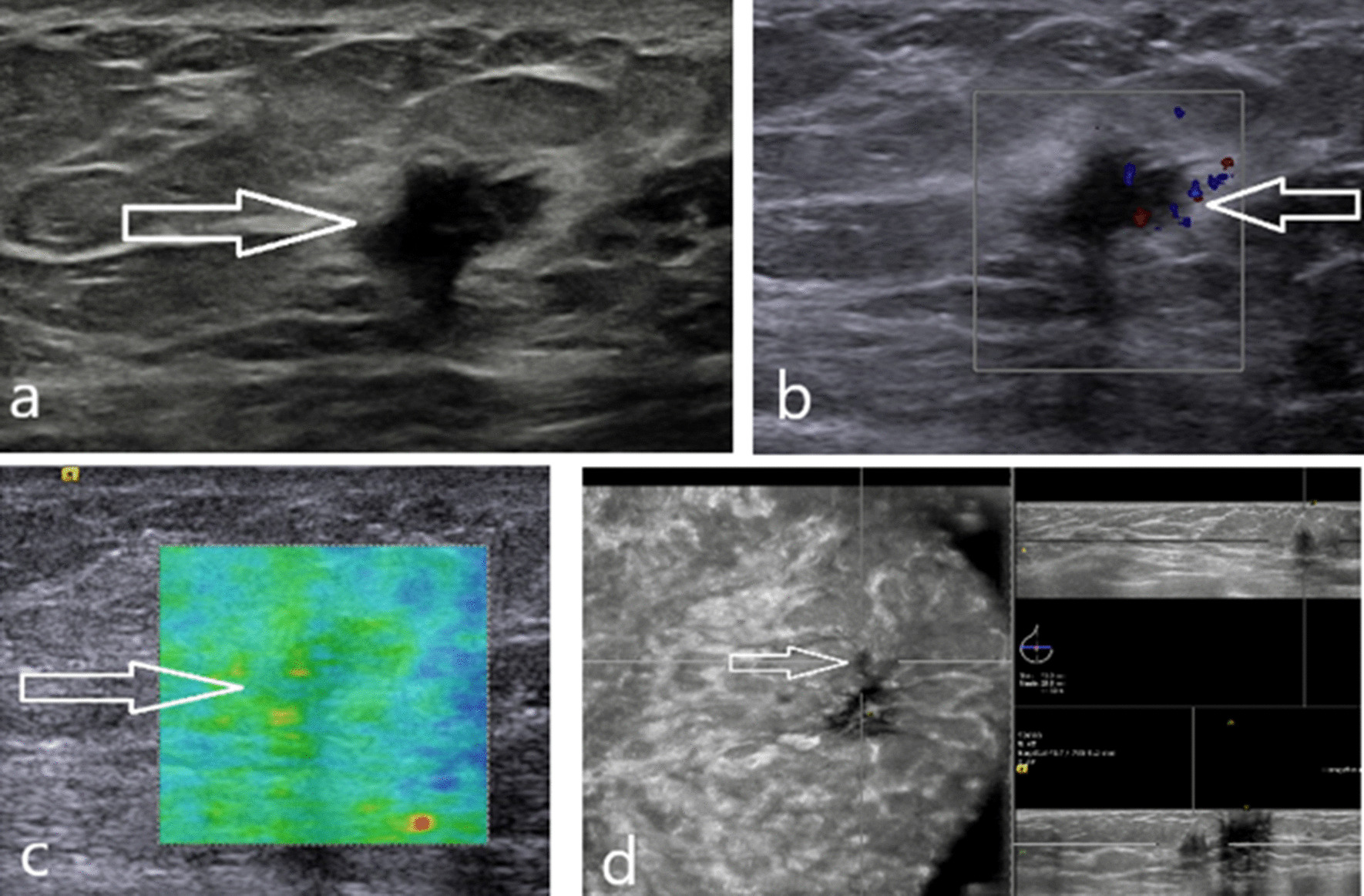
**a** The handheld US revealed an irregularly shaped and markedly hypoechoic nodule with angular edges and an echogenic halo surrounded by subcutaneous adipose tissue and attached to the superficial fascia of the breast (white arrows). **b** The surrounding adipose tissue showed an increased vascular distribution on Doppler US examination (white arrows). **c** Ultrasound elastography showed a Tsukuba stiffness score of 1 of the lesion (white arrows). **d** By ABUS, the lesion showed a retraction pattern on the coronal plane (white arrows)

A 29-year-old woman presented the clinic with a swelling palpable mass in her right breast, which was noticed 6 months before presentation by her. Through examination, the mass was firm, mobile and smooth margin without tenderness to palpation. No palpable axillary and supraclavicular lymph nodes were noted. There was no reported history of breast trauma and breast cancer. The handheld and ABUS examination revealed that a lesion was located 30 mm from the nipple at the 9 o’clock position in the right breast. It was an irregular and markedly hypoechoic nodule measured 9 × 8 × 8 mm, which edges are angular and echogenic halo (Fig. 3). The lesion was almost surrounded by subcutaneous adipose tissue with attachments to the anterior mammary fascia. In addition, the surrounding adipose tissue showed an increased vascular distribution on Doppler US examination (Fig. 3). The elastography revealed a Tsukuba stiffness score of 1 of the lesion. By ABUS (Fig. 3), the lesion showed a retraction pattern on the coronal plane (Fig. 3). Subsequently, US-guided core needle biopsy was performed on the lesion using a 16-gauge needle. Based on the pathological findings, a phyllodes tumor was suspected. The wide excision was then performed, demonstrating a 10-mm area of NF.

## Discussion and conclusions

NF, described for the first time in 1955 by Konwaler et al. [4], is a benign disorder characterized by a pseudo neoplastic proliferation of myofibroblasts. The etiology of NF is uncertain. The clinical progression of NF seems to be a single and rapidly growing lesion [1]. NF can occur anywhere in the body [5]. NF can be divided into subcutaneous, intramuscular and fascial subtypes according to anatomical location. NF can also be classified into three histological subtypes: myxoid, cellular, and fibrous. The same mass may also have different histologic combinations. The different histologic types are roughly correlated with the duration of the nodule [3]. Old lesions are fibrous (such as our first and third cases) while recent cases show myxoid lesions (such as our second case).

Reports of NF in the breast exist but are rare. NF of the breast was listed as one of benign mesenchymal breast tumors by the World Health Organization in 2012 [6]. Although 10–15% of patients have a history of trauma prior to such lesions [3], there was no history of trauma in our three cases. In a previously study, the mean age of the patients with NF of the breast was 39 years (ranging 17–84 years) [7]. Our three cases were conforming to the study. Paliogiannis et al. described that the mean size of the lesion was approximately 20 mm (ranging 6–60 mm) [1], which was also in agreement with our cases. The upper outer quadrants of the breasts were most frequently affected [1], which were not consistent with our first two cases. Our second case was managed within 2 weeks from the first symptoms and signs. This pattern was similar to the previously cases [8]. Such a rapid growth is not common in other breast nodules, especially malignant nodules, and this may indicate even a feature of NF [1].

In Case 2 and Case 3, we provided the ABUS finding of breast nodular fasciitis for the first time. The US of NF may lead to a false impression of malignancy. The US findings may be depend on the histologic characteristics of NF [9]. The lesions that infiltrate into the surrounding fatty tissues make them irregular and unclear boundaries or partial unclear when examined on US. Lee et al. [10] reported that hyperechoicity around the lesion may be associated with marginal interlobular septal fibrosis and infiltrating inflammatory changes. The inhomogeneous hypo-echogenicity may be somewhat related to the uneven distribution of focal keloid-like collagen bundles [10]. The capillary hyperplasia of the lesions make them some vascularity on Dopple US examination.

Since most cases are diagnosed after surgical resection, the natural course of NF is unknown. However, spontaneous regression was observed [11]. Relapse of NF after surgical removal is rare [12]. In Case 1, we provided the longest follow-up time in all literatures. Due to its benign clinical course and limited capacity for recurrence, NF of the breast does not require radical surgical resection. Most patients can be successfully treated with local resection alone, while some may spontaneously resolve without any treatment [13, 14].

In summary, the present three cases demonstrate clinical and US features of NF in the breast. For single, rapidly growing superficial breast lesions, NF can be considered in the differential diagnosis of benign entities resembling malignant tumors on breast imaging.

## Data Availability

Data sharing is not applicable to this article as no datasets were generated or analysed during the current study.

## Abbreviations

NF: Nodular fasciitis
US: Ultrasonography
ABUS: Automated breast ultrasound
